# Sudden Death

**Published:** 1877-05

**Authors:** 


### *SUDDEN DEATH.*

Thought is the stimulus of the brain, too much thought draws too much blood there, and tends to apoplexy in some, madness in others. Thought is the "work" of the brain, sleep is its rest. As thought carries the blood to the brain, sleep gives it vigor, and that passes the blood on, and the brain is rested, and ready for thought again. But if enough sleep is not given, the brain is not ready for work, it has not been sufficiently rested and cleared of its blood, while forced thought increases the quantity; every unrested night adds to the surplus, and apoplexy, which is the sleep of death, or sleeplessness, which is the first step to madness, if protracted, is the ordinary result. It was said of the great historian, that he had an alarm clock to measure the ending of his sleep, and he bounded from his bed at the first stroke. That is, he took it upon himself to say how much sleep his system required, instead of allowing nature to make the appointment, and died prematurely, as all will die who fight against nature. The true plan is to go to bed at a uniform early hour, and rise as soon as nature wakes you up. This simple rule will ordinarily, secure prompt, sound and refreshing sleep, if persisted in, and day sleep is not allowed.

The habit of stimulating the brain to work, when it does not feel like working, is another cause of brain disease, and premature death, and explains why many public speakers die early. They feel under a necessity to speak at an appointed hour, and if they happen not to be "in trim," if the spirit is not upon them, they put the spirit in them, and they speak tea, coffee, opium, tobacco, brandy, "according to the circumstances of the case." Such speaking it is clear, is not from the "natural man," but from the "spiritual," and is not likely to be the "pure milk of the word."

It was the habit of the great Pitt, before "going to the House," to speak in his place, to eat heartily of substantial food, highly seasoned, and then to "swallow a bottle of wine in tumbler fulls," he died of apoplexy at the early age of forty-seven; and those who goad the brain to high activities, whether by tea or tobacco, by coffee or cogniac, will probably meet a similar fate, or if not, a premature death, or worse still, a living death in a mad house.

It is well to note here, how easily a man may cheat himself out of half a life time. The model historian knew that bodily exercise was essential to health, and he took it largely, systematically, on foot and horse, but he did not remember that rest for the brain was quite as necessary to vigorous health as exercise to the body, for he loved to walk and ride alone, with his mind running on his favorite histories, and the brain had no rest, not even the rest of diversion, it was literally worked to death, it had not the power to pass off the blood which continued thought brought there, and its cisterns were broken at the fountain. Let all students therefore remember that "bodily exercise profiteth little," if in the meanwhile, the brain is not rested by diversions, by exercises, as it were, which require no tension, by thoughts without effort.

Newspaper reporters not unfrequently die prematurely. Pleasant is it to take up our favorite paper by a cozy fire of a winter's morning, and have our memories refreshed or our knowledge increased by reading the "report" of a lecture or sermon, or public meeting of the preceding evening, but it is too often the price of blood. Late hours, intense application, a goaded brain and then, in exhaustion, to retire at two or three in the morning, only to have the feverish sleep of over fatigue, the disturbed slumbers which the early noises of a busy city allow, these suffice, with other attendants, to wear out a splendid mind, and a vigorous body, in a very, very few years.

Habitually cold feet occasion brain diseases, by forcing blood there, and keeping it too hot, while they themselves, have not enough. Hence the wisdom of keeping the head cool, and the feet warm. Let it be remembered then, that the body is invigorated by exercise, the brain by rest, and that to goad the brain to action against its instinct, whether by tea, coffee, tobacco, opium or stimulating drinks of any description, will, if persevered in, end in a premature, if not dishonorable death.

---

Most Truthful.—"We too often have occasion to observe that the moral character of an act depends, even in the view of good men, upon the question—who performs it."

